# Mechanisms that Determine the Differential Stability of Stx^+^ and Stx^−^ Lysogens

**DOI:** 10.3390/toxins8040096

**Published:** 2016-03-31

**Authors:** Michael P. Colon, Dolonchapa Chakraborty, Yonatan Pevzner, Gerald B. Koudelka

**Affiliations:** Department of Biological Sciences, University at Buffalo, Buffalo, NY 14260, USA; mpcolon@buffalo.edu (M.P.C.); doloncha@buffalo.edu (D.C.); yonatanp@buffalo.edu (Y.P)

**Keywords:** Shiga toxin, bacteriophage, induction, virulence, RecA

## Abstract

Phages 933W, BAA2326, 434, and λ are evolutionarily-related temperate lambdoid phages that infect *Escherichia coli*. Although these are highly-similar phages, BAA2326 and 933W naturally encode Shiga toxin 2 (Stx^+^), but phage 434 and λ do not (Stx^−^). Previous reports suggest that the 933W Stx^+^ prophage forms less stable lysogens in *E. coli* than does the Stx^−^ prophages λ, P22, and 434. The higher spontaneous induction frequency of the Stx^+^ prophage may be correlated with both virulence and dispersion of the Stx2-encoding phage. Here, we examined the hypothesis that lysogen instability is a common feature of Stx^+^ prophages. We found in both the absence and presence of prophage inducers (DNA damaging agents, salts), the Stx^+^ prophages induce at higher frequencies than do Stx^−^ prophages. The observed instability of Stx^+^ prophages does not appear to be the result of any differences in phage development properties between Stx^+^ and Stx^−^ phages. Our results indicate that differential stability of Stx^+^ and Stx^−^ prophages results from both RecA-dependent and RecA-independent effects on the intracellular concentration of the respective *c*I repressors.

## 1. Introduction

Bacteriophages (phages) are the most abundant biological entities on the planet with approximately 10^31^ individual phage particles [1,2,3,4]. Phages are bacterial parasites whose transmission often results in the death of the bacterial host. Phages cause approximately half of all bacterial mortality [5]. Thus, phages play a crucial role in modulating bacterial populations. 

Upon infecting a host, temperate phages can either grow lytically or integrate into the host chromosome as a prophage. Upon exposure of the host to certain environmental insults, the temperate prophage excises from the host’s chromosome and undergoes lytic growth. The genomes of both cryptic and active temperate prophages are found with surprisingly high frequency in bacterial chromosomes. Since the ultimate goal of phage is to reproduce and, in doing so, these phages kill their host, the prevalence of prophage DNA in bacterial chromosomes is surprising. This suggests that phages are tolerated because their presence provides an evolutionary advantage to the host. 

The Shiga toxin-encoding (Stx) phages are temperate phages that are closely related to the coliphages λ, P22, and 434 [6]. The *stx* genes are located downstream from the P_R_’ promoter in these phages. This promoter is active only during phage lytic growth and, thus, exotoxin is produced only during lytic growth [7,8,9,10,11,12]. Stx is not exported by any bacterial secretory machinery [13,14,15,16] and release of this exotoxin from the bacteria depends on phage genes that cause bacterial lysis [17,18]. Therefore, in the case of Stx-encoding prophages, induction concomitantly allows expression of the *stx* genes [19,20] and those needed for its release.

In all temperate lambdoid phages, the decision to grow lytically or lysogenically relies on the transcriptional regulatory activities of its DNA-bound repressor molecule, *c*I. Induction of a lambdoid prophage requires that *c*I be removed from DNA. The best understood mechanism of induction involves phage surveillance of the health of the bacterial host *via* the bacterial SOS response. The SOS response pathway is responsible for carrying out host DNA damage repair. In this mechanism, the interaction of *c*I with activated RecA (RecA*), the master regulator of the SOS response, stimulates the intrinsic autoproteolytic activity of *c*I. Self-cleavage of this factor dramatically reduces its affinity for specific DNA sites [21,22], de-repressing genes needed for phage lytic growth. RecA-independent mechanisms for inducing lambdoid phages have been identified [23,24,25]. Irrespective of the mechanism, a prerequisite for induction of lambdoid phages is the removal of *c*I from its DNA sites. 

Despite the similarities in the mechanism of lysogen maintenance and induction among all lambdoid prophages, several Stx-encoding prophages have been shown to spontaneously induce at a higher frequency than do related non-toxin-encoding lambdoid prophages [26]. Increased ease of induction has been proposed to contribute to both the virulence and dispersion of the Stx2-encoding phage [14]. Livny and Friedman attributed the increased induction frequency of the Stx-encoding prophages 933W and H19B to a lower concentration of active RecA necessary for induction [26]. Our results suggest that the increased induction frequency of 933W lysogens may be due, at least in part, to a lower amount of repressor in 933W lysogens than in lysogens of the Stx^−^ prophage λ [27]. However, the precise reasons for the relative instability Stx-encoding prophages are not known.

We wish to gain a better understanding of the features of Shiga toxin encoding prophage that underlie their reduced stability/increased inducibility. Knowledge of these features may provide insight into these how these phages evolved to be become both highly prevalent and highly successful in causing human disease*.*

We found that, Stx^+^ prophages BAA2326 and 933W are more likely to induce than Stx^−^ prophages λ and 434. We also found that both RecA-dependent and independent processes contribute to the differences in induction frequency between Stx^+^ and Stx^−^ prophages. This finding suggests that there may be multiple evolutionary causes for the increased instability of Stx^+^ prophages. Interestingly, Stx^+^ and Stx^−^ lambdoid prophages do not differ in their time for maturation or release. This finding suggests that evolution may have selected for Stx^+^ prophages that are first to induce, an adaptation that would allow them to thrive to when the number of new potential hosts is low. 

## 2. Results

### 2.1. Spontaneous Induction of Stx^+^ and Stx^−^ Prophages

To confirm that Stx-encoding prophages are less stable than those of prophages that do not encode Stx, we compared the spontaneous induction frequencies of Stx2-encoding lambdoid prophages 933W and BAA2326 with the Stx^−^ prophages λ and 434. For these experiments, we integrated these phages into MG1655 that either did or did not bear an inactivating mutation in *rec*A [25,28]. 

**Figure 1:**
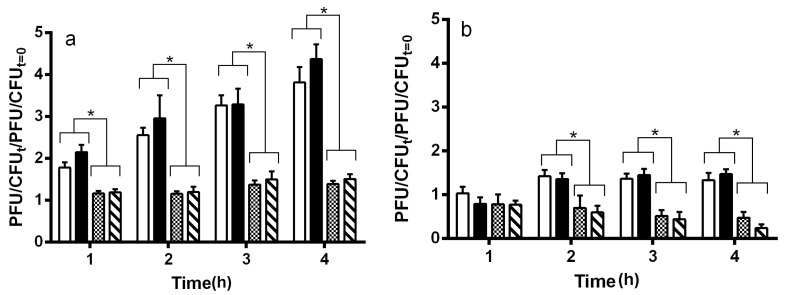
Spontaneous induction of (**a**) MG1655 and (**b**) MG1655 *rec*A mutant lysogens. Wild-type MG1655 (**a**) and MG1655*rec*A (**b**), lysogenized with 933W (white bars), BAA2326 (black bars), λ (stippled bars) or 434 (hatched bars) were grown at 37 °C to mid-log with aeration. Cells were washed and re-suspended in LB supplemented with 1 mM MgSO_4_ and 0.5 mM CaCl_2_. Bacteria and phage were harvested at the indicated time points. At each time point, the amount of phage (plaque forming units —PFU) and bacteria (colony forming units—CFU) were determined as described in the Materials and Methods. Error bars represent ±SEM. The asterisks represent significance at * *p* ≤ 0.05.

Consistent with earlier reports [26], we found that the spontaneous induction frequency of the Stx-encoding prophage 933W is higher than that of the Stx^−^ prophages λ and 434. 434 prophage displayed an identical stability to that of λ (Figure 1a). We found that the spontaneous induction frequency of the Stx-encoding prophage BAA2326 is also higher than that of the λ and 434 prophage. Together, with the earlier findings [26], these results suggest that, as a class, the Stx-encoding phages apparently form less stable lysogens in MG1655 than do naturally-occurring Stx^−^ phages.

Intracellular levels of RecA play a major role in regulating phage repressor levels and, hence, prophage spontaneous induction frequency [23,24]. We wished examine the role of RecA in influencing the differential stabilities of Stx^+^ and Stx^−^ prophages. Consistent with previous findings [23,24,29], *rec*A deletion lowers the overall spontaneous induction frequencies of all prophages examined (Figure 1a,b). Despite the absence of functional RecA, the Stx^+^ prophages still exhibit higher spontaneous induction frequencies than do the Stx^−^ prophages. However, *rec*A deletion decreases the relative differences between the spontaneous induction frequencies of the Stx^+^ and Stx^−^ prophages. Together, the results in Figure 1 show that two mechanisms affect the instability of Stx^+^ prophages 933W and BAA2326 relative to Stx^−^ prophages λ and 434: one that depends upon activated RecA (RecA*) and another that is an intrinsic property of Stx^+^ prophages.

### 2.2. Maturation and Production of Stx^+^ and Stx^−^ Phages 

It is possible that the observed increase in spontaneous induction frequency of Stx^+^ prophage may only be apparent and not a result of a decrease in prophage stability. Differences in the amount of Stx^+^ and Stx^−^ phages produced over time could be the result of variation in developmental characteristics of these classes of phage. In particular, changes in the parameters that affect the maturation time for phage particles and/or the release of newly formed phage could lead to an *apparent* increase in spontaneous induction frequency. To examine this possibility, we measured life history traits of the Stx^+^ and Stx^−^ phages. 

We found that the phage adsorption rates do not differ between the Stx^+^ and Stx^−^ phages we studied (Table 1). Similarly, the Stx^+^ and Stx^−^ phages have virtually identical maturation times, *i.e.*, the eclipse time for all these phages is approximately 25 min and the lysis time for each of these is ~55 min. Consistent with these measurements, we also found that the time it takes for each of these phages to enter the host, reproduce, and lyse the host [30,31] is identical. There are also no significant differences between the phage burst size or the viability (fitness) of the released phage. Together, these measurements indicate that the differences in phage maturation kinetics and/or production do not influence the observed differences in induction frequencies between Stx^−^ and Stx^+^ prophages. 

### 2.3. Effect of DNA Damaging Agents on Stx^+^ and Stx^−^ Lysogens 

**Figure 2:**
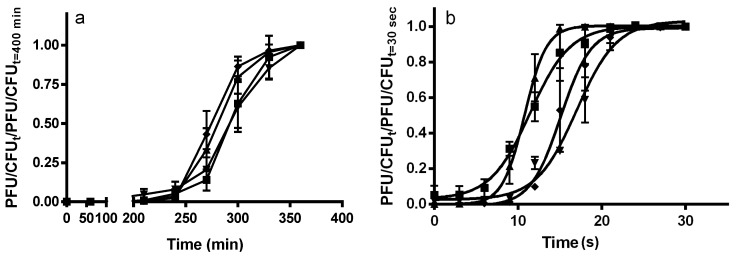
Kinetics of lysogen induction. MG1655 lysogenized with 933W (▲), BAA2326 (■), λ (♦) and 434 (▼) were grown to mid-log phase at 37 °C. The resident prophages were induced by (**a**) adding mitomycin C, or (**b**) irradiating with UV light, as described in Materials and Methods. For samples induced with mitomycin C, the amount of phage (plaque forming units—PFU) and bacteria (colony forming units—CFU) were determined at the indicated times after addition of the agent. For samples induced with UV, samples were irradiated for the indicated time and the amount of PFU and bacteria CFU were determined after 2 h of additional growth. Error bars represent ±standard deviation.

To probe how RecA contributes to the difference in lysogen stability of Stx^+^ and Stx^−^ prophages, we examined the effect of varying the doses of DNA damaging agents: UV irradiation and mitomycin C on induction of these two classes of prophage. Consistent with RecA’s role in stimulating prophage induction, all prophages induced in the presence of these DNA damaging agents (Figure 2a,b). Interestingly, we found that, in the presence of these agents, the two Stx^+^ prophages, 933W and BAA2326, induce more rapidly (Figure 2a) or more readily at lower doses (Figure 2b) than do their two Stx^−^ counterparts. This finding suggests that less RecA* is needed to induce the two Stx^+^ prophages than the two Stx^−^ prophages. To verify that RecA governs the greater sensitivity of Stx^+^ prophages to DNA damaging agents, we repeated these experiments in lysogens formed in MG1655*rec*A. We found that in the absence of functional RecA, the DNA damaging agents had no effect on induction of either Stx^+^ or Stx^−^ prophages (data not shown). 

### 2.4. RecA-Independent Induction of Stx^+^ and Stx^−^ Prophages

**Figure 3:**
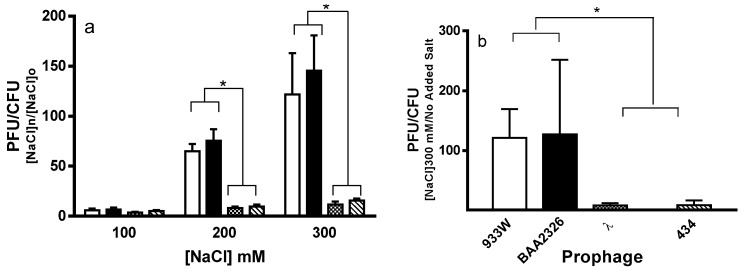
Effect of added NaCl on induction of (**a**) MG1655 and (**b**) MG1655 *rec*A mutant lysogens. Wild-type MG1655 (**a**) and MG1655*rec*A (**b**), lysogenized with 933W (white bars), BAA2326 (black bars), λ (stippled bars) or 434 (hatched bars) were grown at 37 °C to mid-log with aeration. Cells were washed and re-suspended in LB supplemented with 1 mM MgSO_4_, 0.5 mM CaCl_2_, and (**a**) various concentrations of NaCl, or (**b**) 300 mM NaCl. For each condition, the amount of phage (plaque forming units—PFU) and bacteria (colony forming units—CFU) were determined after 5 h incubation as described in the Materials and Methods. Error bars represent ±SEM. The asterisks represent significance at * *p* ≤ 0.05.

**Figure 4:**
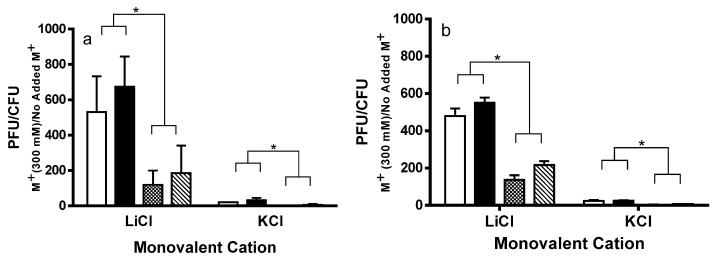
Effect of added various monovalent cations on induction of (**a**) MG1655 and (**b**) MG1655*rec*A mutant lysogens. Wild-type MG1655 (**a**) and MG1655*rec*A (**b**), lysogenized with 933W (white bars), BAA2326 (black bars), λ (stippled bars) or 434 (hatched bars) were grown at 37 °C to mid-log with aeration. Cells were washed and re-suspended in LB supplemented with 1 mM MgSO_4_, 0.5 mM CaCl_2_, and 300 mM LiCl, or KCl. For each condition, the amount of phage (plaque forming units—PFU) and bacteria (colony forming units—CFU) were determined after 5 h incubation as described in the Materials and Methods. Error bars represent ±SEM. The asterisks represent significance at * *p* ≤ 0.05.

To probe the RecA-independent mechanism(s) that underlies the higher induction frequencies of Stx^+^ prophages in the MG1655*rec*A host, we determined the effect of inducers that stimulate prophage induction, but do not activate the SOS pathway. We showed previously that changing the type of cation and/or increasing the cation concentration in the growth medium causes induction of lambdoid prophages [25]. Thus, we examined the effect of added NaCl on the frequencies of induction of Stx^+^ and Stx^−^ prophages. Consistent with our previous findings [25], we found that increased NaCl concentrations in growth medium cause a dose-dependent increase in the frequency of induction of both Stx^+^ and Stx^−^ prophages (Figure 3a). However, the increase in frequency of induction in response added NaCl is greater for the Stx^+^ prophages than as compared to the Stx^−^ prophages (Figure 3a). Similarly, addition of other monovalent cations, (LiCl, KCl) cause both Stx^+^ and Stx^−^ prophages to induce (Figure 4a). However regardless of cation type, the Stx^+^ prophages induce at a higher frequency than do the Stx^−^ prophages (Figure 3a, Figure 4a). The effect of monovalent salts on the differential induction frequencies of Stx^+^ and Stx^−^ prophages is independent of the presence of functional RecA (Figure 3b, Figure 4b). Together with the results in Figure 1b, these data suggests that the decreased stability of Stx^+^ prophages is, in part, an intrinsic property of Stx^+^ prophages.

### 2.5. Intracellular cI mRNA Transcript Levels

**Figure 5:**
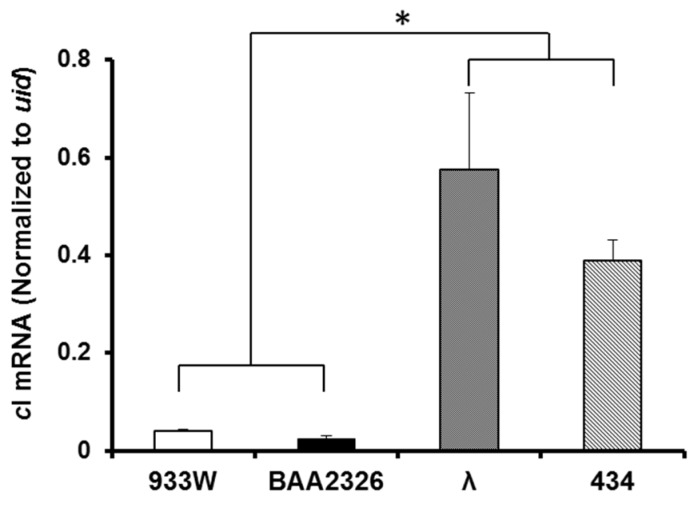
*cI mRNA transcript levels in* *MG1655 lysogens.* Quantitative real-time PCR was used to determine P_RM_ cDNA levels produced from reverse transcription of total RNA extracted MG1655 lysogenized with 933W (white bars), BAA2326 (black bars), λ (stippled bars), or 434 (hatched bars). Amounts of P_RM_ transcripts were normalized to the amount of *uid* transcripts determined in parallel reverse transcription reactions (see Methods and Materials). The amount of P_RM_ transcripts produced by Stx^+^ prophages (933W and BAA2326) are significantly lower (* *p* ≤ 0.01) than those produced by Stx^−^ (λ and 434) prophages.

Maintenance of lysogeny requires that the repressor appropriately occupy its DNA binding sites. Lysogens that contain lower intracellular repressor levels would be anticipated to induce more readily in both the absence and presence of RecA* Therefore we hypothesized that the bacteria lysogenized with Stx^+^ prophage contain lower repressor levels than do those lysogenized Stx^−^ phage. We tested this idea by measuring the amount of repressor mRNA found in bacteria individually lysogenized with the Stx^+^ prophages BAA2326 and 933W or the Stx^−^ prophages λ and 434. We found that the levels of the respective repressor-encoding *c*I transcripts in bacteria bearing Stx^+^-encoding prophage are ~10–30-fold lower than those found in bacteria lysogenized with Stx^−^ phages (Figure 5). This finding is consistent with our *in vitro* transcription results which show that at concentrations needed to fully occupy O_R_2, the repressor partially represses transcription from P_RM_ in 933W and BAA2326 [27,32], whereas under these conditions in 434 and λ, P_RM_ is fully active [33,34]. 

## 3. Discussion

The stability of a bacteriophage lysogen is influenced by host cell physiology. The switch from lysogenic to lytic growth requires inactivation of the repressor’s gene regulatory activity, which normally occurs as a result of it being removed from its DNA binding sites. Our results show that the Stx^+^ prophages 933W and BAA2326 undergo spontaneous induction at higher frequencies than do Stx^−^ prophages 434 and λ (Figure 1a,b). The presence of RecA* and/or the activation of the SOS pathway amplifies the differences between the induction frequencies of Stx^+^ and Stx^−^ prophages. However, the higher spontaneous induction frequency of Stx^+^ prophages is also observed in *rec*A mutant strains, meaning that the observed increased induction frequency of these prophages does not depend solely on RecA*-stimulated repressor autocleavage. These findings are consistent with previous results showing that *rec*A mutant lysogens bearing Stx-encoding prophages release Stx [35,36]. Therefore, the different spontaneous induction frequencies of Stx^+^ and Stx^−^ prophages are driven partly by a RecA*-independent pathway.

Given these findings, two questions remain at issue; (1) what mechanisms cause the higher, RecA-independent, spontaneous induction frequency of Stx^+^ prophage?; and (2) is this higher spontaneous induction frequency a specialized adaptation of this class of phage? Since phage induction requires elimination of the repressor’s gene regulatory activities, one of two ultimate causes of the higher spontaneous induction frequency of Stx^+^ prophages must result from lower repressor occupancy of the operator regions in the Stx^+^ prophages. We envision three mechanisms could cause this decreased occupancy: (1) a decreased DNA binding affinity of Stx^+^ phage repressors for their respective operators, (2) an increased ability of Stx^+^ phage repressors to undergo self-cleavage, and/or (3) a decreased total amount of Stx^+^ phage repressors present in the lysogens [27]. Our work [27,32] indicates that the affinities of the Stx^+^ phage repressors for their DNA sites are similar or higher than the affinities of other lambdoid phage repressors for their cognate operators [37,38,39,40,41]. While we have not directly compared the sensitivity of 933W, BAA2326 , 434, and λ repressors to RecA-mediated autocleavage, our results (Figure 5) indicate that the increased sensitivity of the Stx^+^ lysogens to spontaneous induction is due, at least in part, to a lower amount of repressor in the 933W lysogen than found in λ lysogens.

**Figure 6:**
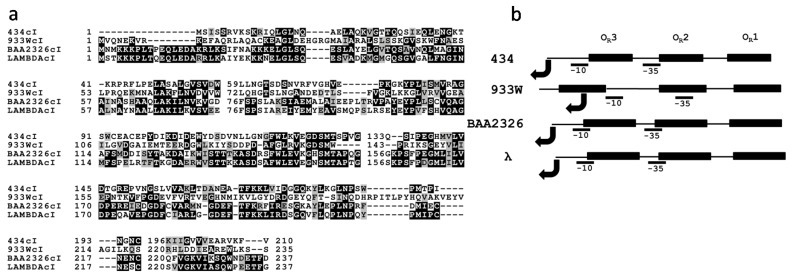
Comparison of (**a**) the repressor sequences and promoter architectures of Stx^+^ (933W and BAA2326) and Stx^−^ (λ and 434) phages. (**a**) Multiple sequence alignments were performed using M-Coffee [47]. Alignment is formatted using BOXSHADE ([48]). Amino acids conserved in two of the four proteins are shaded black. Similar amino acids in two of the four proteins are shaded gray. (**b**) The positions of the operators (boxes) and −35 and −10 promoter elements (underlined) and P_RM_ transcription start sites (bent arrows) in the O_R_ regions of phages 434 [33], λ [43], 933W [46], and BAA2326 [32] are indicated.

Since it appears that the lower repressor concentrations found in Stx^+^ lysogens *vs.* Stx^−^ lysogens is a result of lowered *cI* gene transcription in Stx^+^ lysogens, it is of interest to consider what may lead to lower *cI* transcription in Stx^+^ lysogens. Several possibilities exist. First, since the repressor controls transcription of its own gene in all of the phages studied here [27,32,42,43], it is possible that class-specific differences in the sequences of the repressors lead to the differences in the gene regulatory activities of the Stx^+^ and Stx^−^ phage repressors. However, inspection of the phage repressor sequences (Figure 6a) reveals that the two most closely-related repressors are those of the Stx^+^ and Stx^−^ phages, BAA2326 and λ (61.2% identical/77% similar). In contrast, the sequences of the two Stx^+^ repressors (BAA2326 and 933W) are only 19.3% identical and 34.6% similar and the two Stx^−^ repressors (λ and 434) are 29.4% identical and 46.6% similar. Thus, the sequences of the Stx^+^ phages are not fundamentally different from the repressors of the Stx^−^ phages. Second, class-specific differences in either promoter sequence or structure may affect transcription from P_RM_ in the Stx^+^ and Stx^−^ phages. However, sequence analysis reveals that the P_RM_ promoter sequences of λ, 434, 933W and BAA2326 display equally poor matches to *E. coli* promoter consensus sequences [32,44,45,46]. The juxtaposition of P_RM_ promoter with the respective operators in each phage also does not display class-specific differences between Stx^+^ and Stx^−^ phages (Figure 6b).

In bacteriophages λ and 434, repression of P_RM_ (*via* repressor occupancy of O_R_3) does not occur until after a repressor concentration at which P_RM_ is fully activated (or O_R_2 is maximally filled) is reached. This is because, in these bacteriophages, ≥ 20-fold more repressor is required to fully occupy O_R_3 and thereby fully repress P_RM_ than that needed to occupy O_R_2/maximally stimulate P_RM_ transcription. We previously found that in a 933W lysogen, the 933W repressor, partially occupies its O_R_3 [27], thereby substantially lowering the intracellular steady-state concentration of the 933W repressor-encoding mRNA and presumably the 933W repressor protein. Preliminary results suggest a similar scenario occurs in BAA2326 [32]. Hence, although we have not yet determined whether there are any differences in the stabilities of repressor-encoding mRNAs between Stx^+^ and Stx^−^ lysogens, we suggest that the differences in repressor-encoding mRNA concentration in the Stx^+^ and Stx^−^ lysogens is most likely a consequence of the difference in relative binding affinities of the Stx^+^ and Stx^−^ phage repressors for their cognate sites O_R_; differences that would result in differential P_RM_ promoter activity in the two phage classes. Since a lower repressor-encoding mRNA concentration in the Stx^+^ lysogens would result in lower intracellular repressor concentrations, there should be a greater chance that intracellular repressor levels would fluctuate and fall below the threshold needed to maintain lysogeny, thus explaining the observed higher RecA-independent spontaneous induction frequency of these lysogens. Similarly a lower amount of repressor means that a lower amount of RecA* should be needed to allow prophage induction. This latter assertion is consistent with the finding that Stx^+^ prophages induce more readily at lower doses of SOS inducers. 

It is clear from our data that Stx^+^ lysogens display “hair trigger” inducibility. It is also clear that multiple mechanisms contribute to this “hair trigger” behavior. There are two prevailing and contradicting thoughts regarding prophage inducibility and fitness: (1) that the increased sensitivity to induction decreases prophage fitness [49], and (2) that the increased prophage sensitivity to induction increases Stx^+^ prophage fitness [26]. We argue for the latter. We found that the relative fitness of the two classes of prophages, Stx^−^ and Stx^+^, as well as their absorption rates, eclipse and latent periods and burst sizes are indistinguishable (Table 1). These finding suggest that the developmental characteristics of lambdoid phages are interdependent and have, thus, evolved to an “optimal zone” with each phage exhibiting maximal efficiency for these parameters. These findings further suggest that the greater sensitivity to the environment bestows an advantage to the lambdoid prophage that cannot be obtained from changing the phage’s maturation or production.

Livny and Friedman [26] suggested that Stx^+^ prophages may be subjected to selective pressures not operating on other prophages and that the trait of “higher inducibility” may not provide a selective advantage to the phage during lytic growth. They advanced the notion that it is Stx release that is advantageous (*via* Stx-induced diarrhea and the spread of the non-induced descendants from an original clone) and not necessarily the higher inducibility. We argue it is both. Stx release allows for the survival and advancement via different human symptoms. However, we also argue that the higher inducibility may be an adaption to existence in low host environments. Assuming mass-action kinetics [50], the search time (*t*_s_), in part, would be determined by host density (*N*) and the rate of “finding a host”, which is represented by the absorption rate constant (*k*) [30]. Given that *k* is inversely proportional to lysis time (*t*_L_) [30] and, thus, indirectly to burst size (*b*) [34], empirically represented via ln(*b*)/(*t*_s_ + *t*_L_), and is correlated to phage growth and maturation (assembly), *m*, (*m* = *b*/[*t*_L_ − *e*]) where *e* represents the eclipse period [30,31,51], a change in one physiological characteristic is predicted to impact all phage growth parameters. Given the apparent interdependence and similarity of phage growth parameters (Table 1), how can a phage, in a given environment, become more competitive? We argue that the Stx^+^ prophages have become more competitive by virtue of being able to induce more readily than their Stx^−^ counterparts. More ready induction of Stx^+^ prophages allows for a competitive advantage over other phages in finding a host, particularly in environments where the host density is low.

## 4. Materials and Methods 

### 4.1. Strains and Growth Conditions

Bacterial cultures were grown with agitation either in Luria Broth (LB), or M9 + 0.08% glucose with or without added monovalent salts. Media was supplemented with 50 μg/mL chloramphenicol where appropriate. *Escherichia coli* strains EDL933, BAA2326, and MG1655 were obtained from the American Type Culture Collection (Manassas, VA, USA) The MG1655*rec*A strain, which bears the recA938::*cat* allele, was created by P1 transduction [29]. The Shiga toxin-encoding phages 933W and BAA2326 were obtained by induction of the respective host strains. λ was obtained from a constructed W3110:λ lysogen previously reported [3], and the 434 was from our collection [29]. 

### 4.2. Measurement of Spontaneous and Salt Induction 

Measurement of spontaneous induction and salt induction were previously described [25]. Briefly, cultures of MG1655 or MG1655*rec*A lysogenized with λ 434, 933W, or BAA2326 were grown to saturation overnight in medium + 0.5 mM CaCl_2_ and 1 mM MgSO_4_ at 37 °C. Lysogens were diluted 1:50 in fresh medium + 0.5 mM CaCl_2_ and 1 mM MgSO_4_, grown at 37 °C to exponential phase. To remove any phage particles produced during the overnight growth, the exponential-phase cells were washed three times. Control experiments demonstrated sufficient reduction in the amount of phage particles left from the overnight growth via this methodology. After washing, cells were re-suspended in fresh medium plus 0.5 mM CaCl_2_ and 1 mM MgSO_4_ in a volume equal to the starting culture volume. The suspension medium was either identical to the initial growth medium or it contained varied concentrations of monovalent cations. Lysogens were incubated at 37 °C for various times, up to 5 h. 

To quantify the effects of salt shock and/or spontaneous induction, at the desired time, a portion of “unshocked” and/or salt-shocked cultures were centrifuged at 8000× *g* for five minutes. The phage-containing supernatant was chloroform sterilized. The amount of infectious phage released (plaque forming units—PFU) was determined by plaque assay as described previously [25,52]. An aliquot of the remaining culture was also taken to determine the number of bacteria (colony forming units—CFU) and the amount of infectious phage released per bacterium was calculated as the PFU/CFU ratio. The effect of salt and/or spontaneous induction on phage production was evaluated as an *n*-fold increase determined from the number of phage produced per viable cell incubated in the presence of salt ([PFU/CFU]_Salt_) divided by the number of phage produced per viable cell in the absence of added salt ([PFU/CFU]_No Salt_) or the number of phage produced per viable cell incubated at time point “*t*” ([PFU/CFU]*_x_*) divided by the number of phage produced per viable cell at the initial time point (*i.e.* 0 h) ([PFU/CFU]_initial time_). A *t*-test was used to determine the statistical significance of any changes.

### 4.3. Measurement of DNA Damaging Agents on Prophage Induction

Measurements of mitomycin C induction and UV irradiation induction were performed as previously described [27,49,53,54,55]. Briefly, 5 μg/mL mitomycin C was added to exponentially growing cultures of MG1655 or MG1655*rec*A lysogens and PFU and CFU were measured as described above at designated time points up to six hours. The effect of mitomycin C induction on phage production was evaluated as an *n*-fold increase determined from the number of phage produced per viable cell incubated in the presence of mitomycin C at time point “*x*” ([PFU/CFU]_time *t*_) divided by the number of phages produced per viable cell at time point 6 h ([PFU/CFU]_time point 6 h_). A *t*-test was used to determine the statistical significance of any changes. 

For UV irradiation, lysogens were grown to exponential phase, the cultures cooled to 4 °C, centrifuged, and washed with TMG buffer (10 mM Tris-HCl, -pH 8.0-, 10 mM MgSO_4_, 10 μg/mL gelatin). Bacteria were irradiated by a 254-nm UV light (Mineralight UVG-54, Upland, CA, USA) at a distance of 20 cm (~1 μW/cm^2^) for various times up to 30 s. At each time point, an aliquot was placed on LB plates to determine the number of CFU. Another aliquot was diluted of 1:10 in LB supplemented with 0.5 mM CaCl_2_ and 1 mM MgSO_4._ The aliquot was incubated with agitation at 37 °C for 2 h. The irradiated culture was spun at 18,000× *g*, treated with chloroform, spun again, and the number of PFU determined by plaque assay

### 4.4. Determination of Adsorption Rate

Measurement of individual phage adsorption rates were previously described [30,31,52,56]. Briefly, ~5 × 10^6^ cells/mL of exponentially-grown naïve MG1655 were co-incubated with ~5 × 10^3^ PFU/mL of the respective phage. Aliqouts were removed at specific time intervals up to 10 min and subjected to filter sterilization utilizing a 0.2 micron syringe filter (Corning Life Sciences, Corning, NY, USA). Samples were subjected to previously described plaque assays to obtain PFU/mL counts. The adsorption rates were estimated by fitting the data to the previously described [30] Equation: ln(*P_t_*/*P*_0_) = (−*rN*_0_/μ)(*e*^µ*t*^ − 1), where *P_t_* and *P*_0_ are phage concentrations at times *x* and 0, respectively; *r* the adsorption rate to be estimated; *N*_0_ the bacteria concentration at time 0; and μ the bacterial growth rate.

### 4.5. Determination of Eclipse Period, Latent Period and Burst Size

Measurements for the eclipse period, latent period, lysis time, and burst size were performed as described previously [29,30,31,57,58,59]. Briefly, the burst size was calculated by co-inoculating respective phage particles at a concentration of 1 × 10^7^ PFU/mL with 1 × 10^8^ CFU/mL MG1655 in LB supplemented with 10 mM MgSO_4_ and 5 mM CaCl_2_ for five minutes at 37 °C. The mixture was diluted 10^4^-fold with pre-warmed LB supplemented with 10 mM MgSO_4_ and 5 mM CaCl_2_. At intervals up to 75 min, two 0.2 mL samples were acquired to determine the number of unabsorbed (*U*) phage particles and total (*T*) phage particles. The burst size is calculated as *b* = *F*/(*T* − *U*) where *F* is the final phage count at time point 75 min and *T* and *U* are total and unadsorbed phage, respectively. 

### 4.6. Determination of Phage Fitness

Measurements for fitness were performed as previously described [31]. Briefly, respective phage at a titer of ~2 × 10^3^ PFU/mL were co-inoculated with MG1655 at a concentration of ~2 × 10^5^ CFU/mL in LB supplemented with 10 mM MgSO_4_ and 5 mM CaCl_2_. The mixture was incubated at 37 °C in a tissue culture roller drum and fitness was calculated as *w* = ln(*P*_5_/*P*_0_)/5, where *P*_5_ and *P*_0_ are the free phage concentrations at times 0 and 5 h, respectively.

### 4.7. qRT-PCR

Cultures of the prophage lysogens were grown to late log phase at 37 °C in LB. RNA was extracted from 0.5 mL of cells using the QuickExtract RNA extraction kit (Epicentre, Madison, WI, USA). RNA was further purified by acid phenol extraction followed by chloroform: isoamyl alcohol extraction and precipitation. RNA was re-suspended in DNaseI buffer and RiboGuard, and residual genomic DNA was removed by treatment with RNase-free DNaseI (Epicentre, Madison, WI, USA) for one hour at 37 °C. cDNA synthesis reactions containing purified RNA, Affinityscript buffer, forward primer, and Affinityscript reverse transcriptase/RNase Block enzyme mix (Agilent Technologies, Cedarville, TX, USA) were incubated at 25 °C for five minutes to allow primer annealing, 45 °C for 45 min for cDNA synthesis, and finally at 95 °C for five minutes to heat kill the reverse transcriptase. Quantitation of RNA was performed by real-time PCR. DNA products were detected using Sybr ^®^ Green I (Invitrogen/Molecular Probes Inc., Carlsbad, CA, USA). As an internal control for RNA preparation, separate real-time PCR analyses were performed on the cDNA preparations of the *E. coli uid* gene transcript from the same RNA preparations as the P_RM_ transcripts. The primer sequences used for cDNA synthesis and qPCR are:
BAA2326SenseCGTTACACTCAATCATGTCGAATCAntisenseCCCTGAAGGTATGCTGATACTC933WSense GGGCTAAATTCTTCAACGCTAACAntisense ATCTGTCGCAGACAAGATGG434Sense AGCAGCCAGTCAACACTTACAntisense GGGACTACCCAGCAGTCTATλSenseTCATTGCATGGGATCATTGGGAntisenseCCAGGTGATTTCTGCATAGCCUIDSense GTGTGATATCTACCCGCTTCGCAntisense AGAACGGTTTGTGGTTAATCAGGA

Standard curves for the real-time PCR analysis were made using known template amounts of a plasmid containing the region of interest.

## Figures and Tables

**Table 1:** Bacteriophage Growth Characteristics.

Phage	Adsorption Rate (mL/min)	Eclipse Period (min)	Latent Period (min)	Burst Size	Fitness (h^−1^)
933W	1.64 (0.12) × 10^−9^	0–20	55	159 (12.4)	2.46 (0.15)
BAA2326	1.71 (0.59) × 10^−9^	0–20	55	120 (26.5)	2.21 (0.09)
λ	1.78 (0.73) × 10^−9^	0–20	55	155 (31.1)	2.34 (0.08)
434	1.84 (0.12) × 10^−9^	0–20	55	126 (12.8)	2.66 (0.17)

Parameters characterizing the development and growth of bacteriophage were determined as described in Materials and Methods. Values in parenthesis are ±SEM.
